# Diseases of the Rectum and Anus

**Published:** 1890-03

**Authors:** 


*Diseases of the Rectum and Anus.* Second Edition. By HARRISON CRIPPS, F.R.C.S. Pp. 500. London: J. & A. CHURCHILL. 1890.

We have formed a very high opinion of this work. Based on the double foundation of abundant clinical experience and extended pathological study, the work is, at the same time, the production of a skilful and judicious surgeon. The inclusion of several valuable monographs, the extension and re-writing of several parts, and the introduction of a comparatively novel method of performing inguinal colotomy, bring the work fully abreast with the latest improved doctrines and practices.

The work is essentially personal: the author records his own work and beliefs. Holding as strongly as he does the view (which we to a great extent share) that colotomy by abdominal section is superior to lumbar colotomy, it still strikes us as peculiar that a work dealing with diseases of the rectum and anus has not a word of description to give to the latter operation. The author has certainly the courage to act upon his opinions. We should have liked, too, to have seen some notice taken of certain modifications of the abdominal operation employed by good surgeons, and which we believe would have added to the excellence of the author's. A surgical operation rarely springs forth perfect from the brain of one man. He is a wise inventor who keeps up a full knowledge and appreciation of all other patents bearing on his subject. "Love is blind" in more than one direction; the adage may certainly be applied surgically and mechanically.

